# Climate dependent heating efficiency in the common lizard

**DOI:** 10.1002/ece3.6241

**Published:** 2020-07-16

**Authors:** Alexis Rutschmann, David Rozen‐Rechels, Andréaz Dupoué, Pauline Blaimont, Pierre de Villemereuil, Donald B. Miles, Murielle Richard, Jean Clobert

**Affiliations:** ^1^ School of Biological Sciences University of Auckland Auckland New Zealand; ^2^ Station d'Ecologie Théorique et Expérimentale (SETE), CNRS Moulis France; ^3^ Institut d'Ecologie et des Sciences de l'Environnement de Paris (iEES)‐Paris CNRS, IRD, INRA Sorbonne Université Paris France; ^4^ Centre d'Études Biologiques de Chizé La Rochelle Université CNRS Villiers‐en‐Bois France; ^5^ Department of Ecology and Evolutionary Biology University of California, Santa Cruz (UCSC) Santa Cruz CA USA; ^6^ Muséum National d'Histoire Naturelle (MNHN) Paris France; ^7^ Department of Biological Sciences Ohio University Athens OH USA

**Keywords:** ectotherms, heating efficiency, heating rate, thermoregulation behavior, time budget

## Abstract

Regulation of body temperature is crucial for optimizing physiological performance in ectotherms but imposes constraints in time and energy. Time and energy spent thermoregulating can be reduced through behavioral (e.g., basking adjustments) or biophysical (e.g., heating rate physiology) means. In a heterogeneous environment, we expect thermoregulation costs to vary according to local, climatic conditions and therefore to drive the evolution of both behavioral and biophysical thermoregulation. To date, there are limited data showing that thermal physiological adjustments have a direct relationship to climatic conditions. In this study, we explored the effect of environmental conditions on heating rates in the common lizard (*Zootoca vivipara*). We sampled lizards from 10 populations in the Massif Central Mountain range of France and measured whether differences in heating rates of individuals correlated with phenotypic traits (i.e., body condition and dorsal darkness) or abiotic factors (temperature and rainfall). Our results show that heat gain is faster for lizards with a higher body condition, but also for individuals from habitats with higher amount of precipitation. Altogether, they demonstrate that environmentally induced constraints can shape biophysical aspects of thermoregulation.

## Graphical abstract

In this study, we provide evidence that environmental constraints on thermoregulation shape variation in heating rates among populations. More precisely, we demonstrated that lizards from locations with high rainfall heat faster than lizards from drier sites and that the steady state temperature is positively correlated with the minimal temperature of the environment.

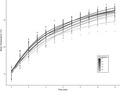

## 1 INTRODUCTION

To reach an optimal body temperature, terrestrial ectotherms require continuous and precise heat exchange between their body and the environment (Angilletta, 2009). Unlike thermoconforming species(whose body temperature mirrors ambient temperatures), species that use behavior to regulate their body temperature rely on external heat sources to optimize heat exchange with the environment (Huey et al., 2012; Sears et al., 2016). Thermoregulating species may use either solar radiation (i.e., heliothermy) or substrate conduction (i.e., thigmothermy) to adjust their body temperature. However, maintaining body temperature in an often narrow optimal range requires constant behavioral adjustments. For example, individuals may shuttle between sun and shade to find appropriate basking sites, or change their activity pattern, body position or posture to make the best use of the surrounding thermal landscape (Angilletta, Cooper, Schuler, & Boyles, 2002; Huey & Kingsolver, 1989). Accurate thermoregulation however entails costs. First, behavioral thermoregulation, by shuttling between basking sites and cooler patches, involves energetic costs related to locomotion (Angilletta, 2009; Huey & Slatkin, 1976; Sears, Raskin, & Angilletta, 2011). Moreover, the time spent basking or shuttling between basking spots limits the time available for other critical activities, such as foraging or social interactions (Angilletta, 2009; Blouin‐Demers & Nadeau, 2005; Huey & Slatkin, 1976). In this context, variation in the thermal environment and fine‐scale spatial heterogeneity may generate spatial heterogeneity in costs associated to behavioral thermoregulation. Ultimately for ectotherms, these costs can affect life‐history strategies and fitness and, in some circumstances, can represent an important selective factor, shaping the expression of individual phenotypes to reduce the cost of thermoregulation in terms of time budget (Gvoždík, 2002).

Costs of thermoregulation can be buffered by adjustments of either behavioral or biophysical mechanisms (e.g., adjusting skin perfusion or coloration). Recent studies have focused mainly on behavioral adjustments of thermal preferences. For example, it has been demonstrated that common lizards (*Zootoca vivipara*) compensate for elevated costs of thermoregulation at higher altitudes by altering their choice of basking sites (Gvoždík, 2002) or thermal preference (*T*_pref_; Trochet et al., 2018). Similarly, it has been demonstrated that the Leόn rock lizard (*Iberolacerta galani*) can seasonally adjust its preferred temperatures to enhance thermoregulation effectiveness (Ortega, Mencía, & Pérez‐Mellado, 2016). Moreover, in a review of 396 species, Clusella‐Trullas, Blackburn, and Chown (2011) demonstrated that thermal preferences of squamate reptiles are negatively associated with local patterns of precipitation and temperature (i.e., species inhabiting sites with limited precipitation favors higher preferred temperatures), suggesting that microhabitat conditions play a role in shaping thermoregulatory behavior. That is, even if the regulation of body temperature in ectotherms involves behavior, different biophysical mechanisms can mitigate the time and energy costs allocated to basking. For example, modulation of an individuals' conductance by increasing skin perfusion leads to faster heat gain in the lizard *Pogona barbata*, whereas reducing it conserves heat over a longer period of time (Grigg & Seebacher, 1999). It is also known that some reptiles can adjust their heart rate and blood flow to increase or decrease heat convection between the surface and core regions (i.e., heating hysteresis; Bartholomew & Tucker, 1963). However, despite a well‐established literature on the physiology of thermoregulation, evidence of direct climatic impacts on intrinsic heating or cooling rates remains rare in the literature. One recent example is the thermal melanism hypothesis: Heating rate depends on skin reflectance, which is also known to covary with elevation or latitude (Clusella‐Trullas & Chown, 2014; Geen & Johnston, 2014; Zeuss et al., 2014). Moreover, some studies indicate that heating and cooling rates can be correlated with altitudinal gradients (Gvozdík & Castilla, 2001; Reguera, Zamora‐Camacho, & Moreno‐Rueda, 2014; Zamora‐Camacho, Reguera, & Moreno‐Rueda, 2014). Nevertheless, the influence of climatic characteristics of the environment per se (e.g., temperature, rainfall or wind) on the variation in biophysical mechanisms remains unclear (Gvozdík & Castilla, 2001) and studies involving multipopulational comparisons are still needed to resolve how climatic conditions and phenotypic characteristics can reduce costs of thermoregulation in ectotherms species.

In this study, we investigated whether environmental conditions are correlated with the rates of heating in the common lizard. This species occurs in a diverse range of thermal environments, making it an ideal model organism to partition the contribution of biotic and abiotic sources of variation in thermoregulation efficiency. We sampled lizards from 10 populations in the Massif Central Mountain range of France and measured the differences in heating rate among a sample of 96 adult males. We then assessed how differences in heating rates of individuals correlated with phenotypic traits expected to influence heat gain (i.e., body condition, dorsal darkness, and initial body temperature; Geen & Johnston, 2014). We also evaluated the correlation between heating rates with the thermal and hydric conditions at each sample site. Previous studies have demonstrated the influence of temperature and rainfall on several morphological (e.g., size, weight, and growth rate; Chamaille‐Jammes, Massot, Aragon, & Clobert, 2006), phenological (e.g., parturition date; Rutschmann et al., 2015), physiological (body temperature, water balance; Dupoué, Rutschmann, Le Galliard, Clobert, et al., 2017; Lorenzon et al., 1999; Dupoué, Rutschmann, Le Galliard, Miles, et al., 2017), or life‐history traits (e.g., litter size; Rutschmann et al., 2016) for this species. Moreover, during the active season, the focal populations inhabit locations that vary from cool microclimates at high elevations, to hot conditions at lower elevation and the species southern range limit (Bestion, Teyssier, Richard, Clobert, & Cote, 2015). Therefore, we hypothesized that the high sensitivity to temperature combined with a major climatic contrast should lead to natural selection favoring the reduction in costs of thermoregulation in stressful environments. We predict differences in the average heating rate among populations: Heat gain should be faster in lizards occupying cool habitats, but slower in individuals from warm habitats.

## 2 MATERIALS AND METHODS

### 2.1 Study species

The common lizard (*Z. vivipara*) is a small‐sized species in the family Lacertidae whose distribution extends throughout Europe and Asia. At our study localities, females are viviparous and gestation is concurrent with our study (Bleu et al., 2013). Pregnancy in lizards is known to alter a female's thermoregulation behaviors by affecting body shape and preferred body temperature (Le Galliard, Le Bris, & Clobert, 2003). Therefore, we focused on males to avoid any indirect effects linked to female gestation. Males emerge from hibernation in late April and immediately explore their habitat to search for females. After mating, males accumulate energetic reserves to recover from reproductive activity until entering hibernation in late autumn. The common lizard is considered to be an efficient thermoregulator, coping with variation in its thermal environment (among or within populations) by adjusting notabely the frequency of basking (Gvoždík, 2002). In our study system, the activity period for lizards is between 0900 and 1800 hr and we have observed males basking or foraging on a variety of substrates including rocks, trees, bushes, or moss; all typical microhabitats of the humid peat bogs inhabited by common lizards.

### 2.2 Lizard capture, morphological measurements, and dorsal coloration

In this study, we sampled 96 individuals from 10 populations between 19 and 28 June 2015. At each location, we captured approximately 10 males and brought them to our breeding facilities, in Villefort (Lozère, France). We measured snout to vent length (SVL; mean + *SE*=54.4 ± 2.9 mm), body mass (3.25 ± 0.55 g), and dorsal darkness (DD, see details below). Body condition (BC) was estimated as the residuals of a linear regression between body mass (response variable) and SVL (predictor variable; see Appendix S1 for more details; Richard et al., 2012). After capture, lizards were housed in individual plastic terraria (12 cm × 18 cm × 12 cm; Massot & Clobert, 2000) until we assessed their heating rate (two days after capture). Each terrarium contained a cardboard shelter, was layered with heather earth, and misted three times a day to maintain a mesic environment. Lizards were exposed to daylight, but they were not provided heat source to avoid acclimation to the artificial heat source (30W Bulb) provided for thermoregulation in our breeding facilities. Lizards were not fed between capture and the experiment to maintain them all in the same digestive state. Immediately after the experiment, they were fed ad libitum with crickets (*Acheta domesticus*) and misted with water. All animals were released at the site of capture at the end of our field season.

Proportion of dorsal darkness (DD) has already been demonstrated to be a good proxy of dorsal melanism and was computed following the methodology described in Jacquin et al. (2011). We obtained digital images of the dorsum for each male by scanning with a high‐resolution computer scanner (Canon^®^, CanoScan Lide 110; image size: 2,550 × 2,600 pixels). We gently held the lizard motionless by placing a patch of soft, black moss on the ventrum of the animal. Digital images were stored as.*png* files. Our method of image acquisition reduces potential biases in the estimates of dorsal darkness due to differences in luminosity between pictures. Using ImageJ software (Schneider, Rasband, & Eliceiri, 2012), we selected a section of each individuals' back, going from the fore to the hind legs, and transformed it into a 32‐bit black and white picture. The score of dorsal darkness was calculated as the proportion of black pixels in this section by using the grayscale threshold option.

### 2.3 Habitat characterization

**FIGURE 1:**
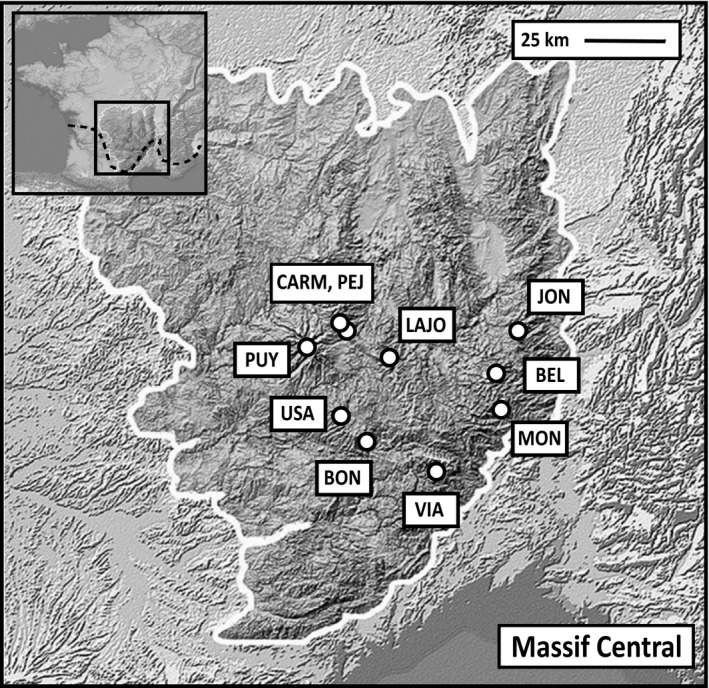
Location of the common lizard populations sampled in this study (Massif Central, France). The dashed line represents the Southern margin of the species distribution. Localities of the populations: BEL (Bel Air), BON (Col de Bonnecombes), CARM (Carmantran), JON (Mont Gerbier de Jonc), LAJO (Lajo), MON (Tourbières de Monstelgues), PEJ (Pejouzou), PUY (Puy Mary), USA (Usanges) and VIA (Tour du Viala), see Table 1 for additional details

Our study populations were located in the Massif Central mountain chain, at the southern distribution limit of the common lizard (Figure 1). To maximize our chances to sample populations from different thermal and hydric characteristics, study sites were chosen over the spatial extant of the Massif Central region (maximal distance between two sites = 117 km; minimal distance between two sites = 1 km) to: (a) span an elevational gradient from 1,050 to 1,500 m asl and (b) obtain different representative habitat types colonised by the common lizard. To estimate the gradient in thermal conditions among capture localities, we recorded air temperature (*T*_a_) at each site every 30 min, using 2–3 iButton temperature data loggers (Thermochrons^©^; Maxim Integrated Products,) as described in Dupoué, Rutschmann, Le Galliard, Clobert, et al. (2017). Each data logger was placed in a small ‘T’ shaped PVC container to protect it from direct insolation, rain or curious animals, while allowing airflow. We placed the sensors on the ground and in the shade of vegetation in microhabitats observed to be used by lizards at each site (e.g., rush, *Juncus* spp., heather, *Calluna vulgaris*, or blueberry shrubs, *Vaccinium myrtillus*). These measures are different from operative temperatures as they do not integrate the effect of solar irradiance or wind speed (Dzialowski, 2005). However, in this study we chose to focus on studying the effect of common climatic variables used to describe ecological responses of the common lizard to its biophysical environment (Chamaille‐Jammes et al., 2006; Dupoué, Rutschmann, Le Galliard, Clobert, et al., 2017; Rutschmann et al., 2016). We simultaneously recorded temperatures from all populations during a period of 21 days (from 29 June to 17 July 2015), starting on the day of capture. We used two temperature variables to describe each site: *T*_Hmax_ and *T*_Hmin_. We also estimated the average amount of rainfall for each habitat (*R*_hab_) using records from the nearest weather station of the French meteorological agency (Meteo France) over the same period. We chose to use two different temperatures (*T*_Hmax_ and *T*_Hmin_) as they differ in their impact on ectotherm ecophysiology (Dupoué, Rutschmann, Le Galliard, Clobert, et al., 2017). The minimal temperature of the habitat *T*_Hmin_ portrays nocturnal temperatures that common lizards cannot avoid by behavioral means, for example, microhabitat selection. In contrast, the daily maximal temperature of the habitat *T*_Hmax_ is measured at the thermal peak of the day and provides a more accurate estimate of the behavioral constraints on activity and the potential risk of overheating. Finally, note that due to their spatial proximity, some populations share the same values of precipitation. Study sites exhibited large differences in their thermal characteristics (*T*_Hmin_: average minimal temperature = 9.7°C, range – 8.0 to 12.6°C; *T*_Hmax_: average maximal temperature = 32.7°C, range – 22.7 to 39.2°C) and precipitation rates (*R*_hab_: average precipitation rate = 1.3 mm/day, range – 0.21 to 2.5 mm/day), see Table 1 for additional details.

**TABLE 1:** Location of the study populations and associated climatic descriptors are also provided the average daily minimal temperature (*T*_Hmin_), daily maximal temperature (*T*_Hmax_), daily precipitation rate measured over 3 weeks (from 29 June to 17 July 2015) and elevation

Population	Code	Coordinates	*T*_Hmin_ (°C)	*T*_Hmax_ (°C)	Precipitation (mm/day)	Elevation (m)
Belair	BEL	44°40′21.04″N 4°1′31.67″E	8.80 ± 0.56	33.96 ± 0.49	0.21 ± 0.21	1,463
Col de Bonnecombes	BON	44°33′38.73″N 3°7′40.36″E	9.73 ± 0.12	24.46 ± 0.51	2.12 ± 0.61	1,397
Carmantran	CARM	45°9′25.05″N 2°50′18.78″E	9.68 ± 0.92	32.97 ± 0.79	1.98 ± 1.11	1,267
Gerbier de Jonc	JON	44°50′30.12″N 4°12′52.70″E	10.06 ± 0.43	33.73 ± 0.68	0.54 ± 0.34	1,385
Lajo	LAJO	4°50′40.23″N 3°25′49.90″E	8.79 ± 0.46	39.04 ± 0.66	1.52 ± 0.72	1,382
Montselgues	MON	44°30′40.26″N 4°00′29.36″E	11.42 ± 0.42	33.13 ± 0.32	0.20 ± 0.19	1,099
Pejouzou	PEJ	45°09′52.24″N 2°50′37.52″E	8.00 ± 0.95	39.19 ± 1.04	1.98 ± 1.11	1,254
Puy Mary	PUY	45°6′25.77″N 2°41′6.10″E	12.63 ± 0.60	32.55 ± 0.84	2.50 ± 1.42	1,470
Usanges	USA	44°38′44.87″N 3°7′32.50″E	9.02 ± 0.27	22.68 ± 0.49	2.12 ± 0.61	1,273
Tourbiere du Viala	VIA	44°20′17.43″N 3°46′04.76″E	8.53 ± 0.52	35.45 ± 0.58	0.14 ± 0.12	1,190

### 2.4 Heating rate: Experimental design

To assess heating rate, each individual was placed in a terrarium within a temperature‐controlled environmental chamber set to 5°C, which is close (but above) the species critical thermal minimum. We kept lizards within the chamber for 15 min. Lizards were removed from the environmental chamber and allowed 5 min of acclimation in a light gray PVC tube (diameter: 8 cm; height: 15 cm), at the ambient laboratory temperature (*T*_lab_; 25.3 ± 1.2°C). Lizards were then exposed to a 30 W incandescent bulb (range of wavelength: 350–800 nm) suspended 15 cm above the chamber. Dorsal skin temperatures were recorded every 60 s for 15 min using an infrared thermometer (model Fluke^®^568) focused on the dorsal area. The distance of detection (150 mm) was calibrated between all measures according to the manufacturer recommendations for a spot size of 0.9 mm (smaller diameter; distance‐to‐spot ratio is 50:1). Skin temperature recorded with infrared thermometer is known to correlate with core body temperature in small lizards and thus considered a good proxy of it (Artacho, Jouanneau, & Le Galliard, 2013; Gilbert & Miles, 2019). However, to confirm this assumption, we measured this relationship independently from our experiment and found a significant correlation (Pearson product–moment correlation – *r* = .98; *t* = 75.98, *df* = 87, *p* < .005; see Appendix S2 for further details).

The temperature of the PVC tube (*T*_tube_) was also recorded at the beginning of each experiment, prior to introducing the lizard (*T*_tube;_ 31.0 ± 1.5°C). We then monitored temperatures every minute over 15 min. Because several lizards were run at the same time, it was too complicated to standardize initial body temperature for all lizards and then start to measure heating rates for each lizard. Consequently, we decided to set the initial temperature (*T*_init_) for each individual as the body temperature closest to 10°C (*T*_init_ ± *SD* = 10.5 ± 1.3°C), which, in average, corresponded to the second or third measurement for each animal. We then used the following 12 measurements of temperature in our analyses (e.g., if the closest measurement to 10°C was the second one, we only retained measurement 2–14). This allowed us to compare heating rate dynamics over the same period and from similar initial body temperature across all individuals.

### 2.5 Heating rates: Mathematical characterization

**FIGURE 2:**
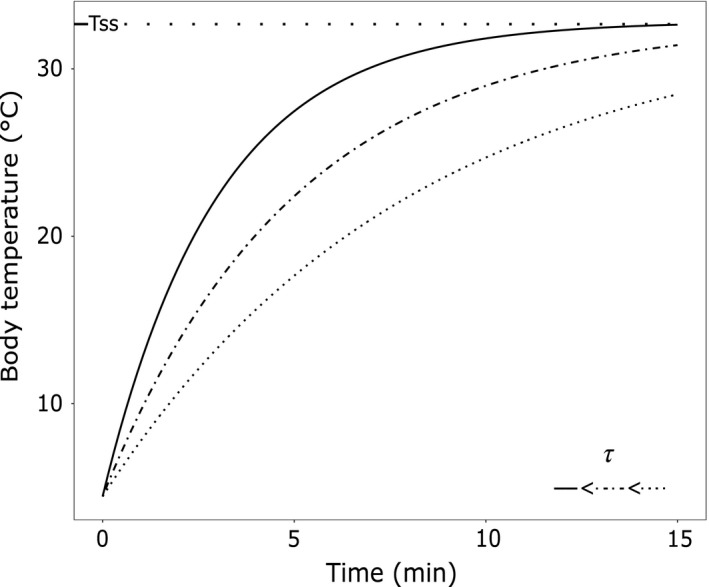
Illustration of the mathematical functions used to describe heat accumulation. Heat curve function is represented for a set of three different values of *τ* (in ascending order: dotted line, dashed line, solid line) and with a constant *T*_ss_ (32°C). Note that a high value of *τ* indicates a slow rate of temperature gain

We used the heating curve for ectotherms as described in Bakken and Gates (1975), to determine the heating rates for each individual and investigate potential differences among populations (Equation 1; Figure 2):

(1) $$T_{\left(t\right)}=T_{\text{ss}}+\left[T_{\text{init}}-T_{\text{ss}}\right]e^{-t/\tau }$$

where *T*_(_ *_t_*_)_ represents the body temperature at time *t, T*_ss_ the steady‐state temperature, *T*_init_ represents initial body temperature, and *τ* the heating rate. This was also used by Gvoždík (2002) in an earlier study on the common lizard. Faster heating rates would result in smaller values of the heating rate (*τ*). The steady‐state temperature (*T*_ss_) is not directly measured on individuals but is fitted from the statistical model, as the extension of each individual's heating curve (see Figure 2 for a graphical representation). Also note that *T*_ss_ as estimated in our experiment slightly differs from *T*_e_ (operative temperature) assessed in Gvoždík (2002), probably because in our design lizards were not immobilized, but had limited freedom of movement.

### 2.6 Heating rates: Statistical analyses

The statistical models were constructed using the *nlme* function (package nlme, Pinheiro et al., 2018) in R.3.4.3 (R Core Team, 2018). This function fits a nonlinear mixed‐effects model to the data (here, the nonlinear model is based on Equation 1) and has the ability to include nested random effects. Accordingly, the function estimates the thermal time constant for heating (*τ*) and the steady‐state body temperature (*T*_ss_) for each individual and simultaneously tests the relative influence of a given set of parameters on their computation. In other words, covariates are included in estimating the heating rates parameters. In the fixed effects part of the model, we tested the influence of body condition (BC), dorsal darkness (DD), tube temperature (*T*_tube_), initial body temperature (*T*_init_), habitat temperature (*T*_Hmin_ and *T*_Hmax_), elevation (Elev), and rainfall (*R*_hab_) on *T*_ss_ and *τ* estimates. We also included all interactions of biological interest (i.e., BC × DD, *T*_0_ × DD, *T*_Hmin_ × R_hab_, *T*_Hmax_ × *R*_hab_). However, none of the interactions were significant, so we have not presented their values in the manuscript. Because there are multiple temperature measurements for each individual, and because of the nonindependence of individuals within populations, we included individual ID, nested within population, as a random effect. The significance of fixed effects were assessed using backward selection of nonsignificant terms (threshold of significance *p*‐value <.05, Zuur et al., 2009). To facilitate convergence, all continuous variables were mean‐centered and scaled by their standard deviation.

## 3 RESULTS

**FIGURE 3:**
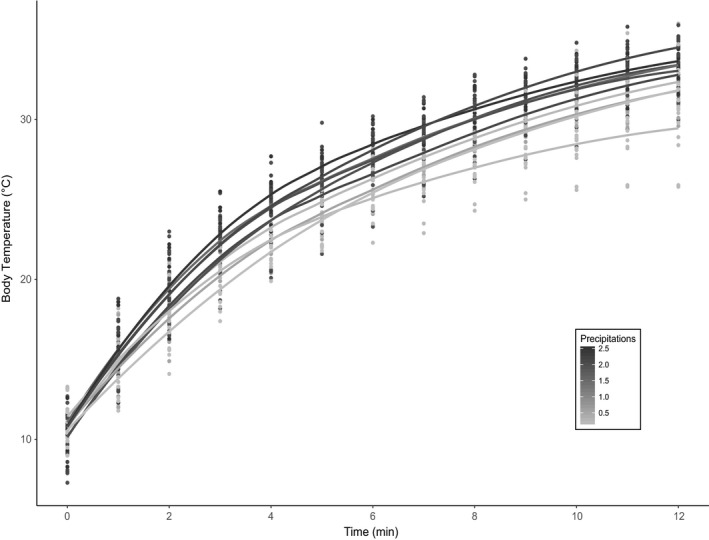
Heat gain versus precipitation among population. Representation of skin surface temperature according to precipitation rate in the studied populations. Dots represent individual measures. Solid lines represent an average heat curve for each population. A scale of precipitation rates is provided in gray: The darker the coloration, the dryer the environment

The heating rate experiment revealed a positive correlation between the thermal time constant for heating (*τ*) and body condition (BC); slender lizards heated up faster than lizards with a high mass per unit of length (the smaller *τ*, the faster the heat rate; Table 2). We did not detect an effect of dorsal darkness on *τ*. We found that only rainfall influenced *τ*, with higher precipitation associated with a faster heating rate (Figure 3). None of the remaining environmental variables (temperature or elevation) were retained in our models. The correlation coefficient between *T*_Hmin_ and *T*_Hmax_ is low (0.16), justifying inclusion of both variables in the analysis. However, we found no influence of environmental temperature (*T*_Hmin_ or *T*_Hmax_) on heating rates.

**TABLE 2:** Summary of nonlinear mixed effect models relating the thermal time constant for heating (*τ*), and steady‐state body temperature (*T*_ss_) versus. initial body temperate (*T*_init_), tube temperature (*T*_tube_), body condition, dorsal darkness, habitat rainfall (*R*_hab_), temperatures (*T*_Hmin_, *T*_Hmax_) and elevation (Elev). Note the smaller value of *τ* corresponds with a faster rate of heating. Individuals were nested within population as a random effect

		Predictor variables	Estimate	*SE*	*df*	*t*‐Value	*p*‐Value
Heating rate	*Τ*	Intercept	6.63	2.8	1,135	2.34	<.001
		*T*_init_	−0.05	0.07	1,135	−0.62	.53
		*T*_tube_	0.08	0.07	1,135	0.97	.33
		Body condition	1.10	0.27	1,135	3.99	<.001
		Darkness	0.01	0.01	1,135	1.03	.29
		*R*_hab_	−0.54	0.13	1,135	−3.96	<.001
		*T*_Hmax_	−0.03	0.02	1,135	−1.36	.17
		*T*_Hmin_	−0.09	0.07	1,135	−1.32	.18
		Elev	−0.001	0.001	1,135	−0.77	.44
	*T*_ss_	Intercept	21.8	6.32	1,135	3.45	<.001
		*T*_init_	−0.01	0.17	1,135	−0.07	.94
		*T*_tube_	0.51	0.18	1,135	2.91	.004
		Body condition	0.75	0.61	1,135	1.22	.22
		Darkness	0.03	0.02	1,135	1.02	.31
		*R*_hab_	0.16	0.30	1,135	0.54	.58
		*T*_Hmax_	−0.03	0.04	1,135	−0.81	.41
		*T*_Hmin_	−0.47	0.16	1,135	−2.86	.004
		Elev	0.001	0.002	1,335	0.75	.45
			***SD***	**Corr**			
Random effects (Id in Pop)		*τ* (intercept)	0.58				
		*T*_ss_ (intercept)	1.33	0.63			
		Residuals	0.66				

Lizards exhibiting higher body temperatures at the end of the experiment also had high steady‐state temperatures (see Appendix S3). *T*_ss_ was not influenced by any of the phenotypic traits. Regarding the influence of habitat, we did not detect any effect of precipitation nor elevation; but our results revealed a significant negative correlation between *T*_ss_ and the minimal temperature of the habitat (*T*_Hmin_). The correlation coefficient between *T*_Hmin_ and *T*_Hmax_ for this model was low (0.18), and both variables were retained in the model. Tube temperature positively influenced *T*_ss_: The warmer the tube, the warmer the lizard at the end of the experiment.

## 4 DISCUSSION

### 4.1 Individual phenotypes and heating rate

Of the two phenotypic traits included in our study, only body condition influenced the dynamic of heating rates: Individuals with a higher body condition index (BC) have a lower heating rate. This result is not surprising as a large body mass could decrease the velocity of heat gain (Stevenson, 1985; Zamora‐Camacho, Reguera, & Moreno‐Rueda, 2014). Body condition of the common lizard has been demonstrated to significantly increase when individuals were experimentally exposed to warmer temperature in enclosures (Bestion et al., 2015), a pattern already observed in natural populations from the Massif Central (Chamaille‐Jammes et al., 2006). Along with our results these observations show that an increase in BC in populations of common lizards could lead to slower heating rates, potentially (a) reducing the time budget of larger individuals for foraging and mating and (b) counterbalancing the risk of overheating associated with warmer temperatures. More studies are needed to understand how a slower heating rate will impact wild populations of small ectothermic species. However, recent studies have suggested that increased body condition is related to a higher probability of population extinction in the common lizard, suggesting that slow heating rates cannot completely counterbalance overheating issues (Bestion et al., 2015; Sinervo et al., 2010).

Our results did not support the color‐mediated thermoregulation hypothesis (or thermal melanism hypothesis). That is, darker individuals should heat faster than lighter ones, as their absorbance of radiant energy is higher (Bartlett & Gates, 1967; Geen & Johnston, 2014; Umbers, Herberstein, & Madin, 2013). This hypothesis has already been experimentally tested and confirmed for other ectotherms (Gibson & Falls, 1979; Geen & Johnston, 2014 but see Bittner, King, & Kerfin, 2002), including the common lizard (Bestion et al. *unpublished data*). The absence of this relationship in our system therefore remains an open question. One likely explanation would be that the influence of melanistic coloration differs according to body size, body shape, or body condition (Bartholomew, 1981; Geen & Johnston, 2014; Matthews et al., 2016). Indeed, smaller or slender individuals should have higher heating rates due to a high surface area to volume ratio and are therefore expected to have higher reflectance to counterbalance the increased heating rate. However, we did not detect a statistically significant interaction between body condition and dorsal darkness in our models, suggesting that we may not have capture enough variation in dorsal darkness in our sample size.

### 4.2 Environmental conditions and heating efficiency

A significant outcome of this study is the demonstration that the biophysical properties of heating rate can be adjusted according to environmental conditions: We detected faster heat gain for lizards occupying habitats with higher amount of precipitation. The most likely explanation is that rapid body warming in rainy environments allows lizards to make the best use of scarce thermoregulation opportunities and save valuable time for feeding or mating. However, effects of precipitation are more complex, because low precipitation rates are not only correlated with more frequent opportunities of basking, but also represent a potential constraint on water balance (Ladyman & Bradshaw, 2003; Mautz, 1982; Rozen‐Rechels et al., 2019). Habitats with low rainfall may induce chronic dehydration, impose more extensive restrictions on basking because of evaporative water loss, and lead individuals to target lower body temperatures to conserve water (Anderson & Andrade, 2017; Dupoué et al., 2015; Köhler et al., 2011). Concomitantly, higher amounts of precipitation relax constraints on water conservation and targeting higher body temperatures is less risky. In a mesic environment, the benefits of rapid rates of heating are important, because lizards are capable of reaching higher body temperatures sooner with fewer costs related to water balance (Angilletta, 2009).

Contrary to our expectation, we found that the heating rate *τ* was influenced solely by precipitation and not by temperature. To better appreciate this result, it is necessary to understand how temperature and precipitation covary within, but also among populations in our study system. Despite the existence of inter‐population fluctuations in temperature, we suggest that intra‐site variation is as important (if not more) as variation among sites. Indeed, the average minimal and maximal temperatures recorded by the iButtons over the 21 days of sampling, ranged from 8°C to 12.63°C and from 22.68°C to 39.19°C for *T*_Hmin_ and *T*_Hmax_, respectively. In comparison, the range of temperature observed within the coldest population (Usanges) can extend from 8.5°C to 40.5°C within 24 hr, a breadth of ambient temperatures offset by efficient modifications of the thermoregulatory behavior of the common lizard (Gvoždík, 2002). On average, thermal conditions may be similar enough among study sites to be buffered by behavioral adjustments, thus preventing the necessity of physiological adaptations of heating rates. The effect of rainfall, however, is less likely to be buffered by modifications of thermoregulatory behavior. The site with the lowest rainfall (VIA) was characterized by a total amount of precipitation of 4.2 mm over the 3 weeks of sampling. Over the same period, the population with the heaviest rain (PUY) received almost 18.5 times more rain (77.6 mm). Note that the average daily precipitations that we measured over 3 weeks are well correlated with the conditions measured during the past 20 years (Appendix S4). It is therefore likely that the long‐term trends of rainfall observed in each habitat constrain a heliothermic species such as the common lizard (i.e., by forcing individuals to retreat into their refuge when rain and disrupting any chance of basking or foraging) and shape its evolution. Although the magnitude of rainfall fluctuates among our study sites, it is reasonable to assume that basking opportunities and water balance constraints exhibit large differences among our study sites, creating a need for efficient heat exchange mechanisms where basking opportunities are scarce or where a humid environment does not balance risks of overheating.

We also detected a direct effect of environmental temperatures on equilibrium body temperatures (*T*_ss_). Although *T*_ss_ is not directly linked to heating velocity, it does provide information about heating efficiency: The higher the *T*_ss_, the higher the lizard's body temperature after 12 min (see Appendix S3; Gvoždík, 2002). Specifically, our results show that lizards inhabiting sites characterized by warmer minimal temperatures (*T*_Hmin_) had lower body temperatures at the end of the heating experiment. Minimal temperatures are usually recorded at night and the relationship with thermoregulation efficiency can therefore be surprising at first glance. One potential explanation is that the cold adapted common lizard uses night as a period of physiological rest. Although nocturnal behavior is relatively unknown in this species, multiple focal observations suggest that individuals spend the night sheltered in vegetation or buried in peat moss close to the surface (Grenot et al., 2000). When nocturnal temperatures increase, lizards must therefore endure the warmer *T*_Hmin_, as opportunities for behavioral adjustment to buffer them are scarce (e.g., switching nocturnal retreat sites overnight is unlikely). Warmer conditions are therefore likely to induce an increase in resting metabolic rate. If substantial enough, this abnormal intensification of physiological activity level could generate a state of metabolic meltdown (Huey & Kingsolver, 2019) leading to a reduced lifespan and an eventual population collapse as demonstrated for the common lizards in our study area (Dupoué, Rutschmann, Le Galliard, Clobert, et al., 2017). Consequently, lizards exposed to physiological stress by warmer nocturnal conditions may be using other biophysical adaptations to prevent deleterious effects of any additional source of metabolic stress, for example, their upper thermal tolerance limit. In other words, a lower heating rate could be advantageous in preventing excessive physiological stress. This explanation is however hypothetical and further work needs to be done to explore the effect of nocturnal global warming and confirm its effect.

## 5 CONCLUSIONS

In this study, we focused on understanding how individual variation in heating rate among different populations of the common lizard could compensate for heterogeneity of the thermal landscape and ameliorate the costs of thermoregulation. Our results demonstrate that the efficiency of heating rates can be affected by both individual phenotypes (body condition negatively correlates with heating rates) and environmental factors (precipitation rates negatively affect heating rates while minimal temperatures negatively correlate with steady‐states temperatures). However, once at their optimal temperature, individuals also have a direct interest in maintaining a stable body temperature as long as possible to take advantage of an extended activity period. In parallel to this study, we also explored the impact of climatic conditions on cooling rates. Our results suggested that individuals from colder habitats are more effective at maintaining their body temperatures (Appendix S5). However, some methodological issues exist in this experiment and more work is required to confirm this aspect.

Our work highlights that time and energetic restrictions are not the only constraints shaping biophysical properties of thermoregulation. Indeed, the physiological costs imposed by warm microclimatic conditions on water or energy balance may represent another set of selective pressures. Our work also suggests that the variation we observed in thermoregulation efficiency is likely to result from an adaptation to differences in long‐term abiotic condition among the sample sites. Additional experiments including common garden or reciprocal transplants could help depict the selective advantages of thermoregulatory efficiency in the context of global warming.

## CONFLICTS OF INTEREST

None declared.

Permits: Permission to capture and handle lizards was provided by the ‘Office Nationale des Forêts’, the ‘Parc National des Cévennes’, and the regions Auvergne, Rhône Alpes, and Languedoc Roussillon.

## AUTHOR CONTRIBUTIONS

**Alexis Rutschmann**: Conceptualization (lead); data curation (lead); formal analysis (lead); methodology (lead); project administration (equal); visualization (lead); writing – original draft (lead); writing – review and editing (lead). **David Rozen‐Rechels**: Conceptualization (equal); validation (equal); writing – original draft (equal); writing – review and editing (equal). **Andréaz Dupoué**: Conceptualization (equal); data curation (equal); validation (equal); writing – original draft (equal); writing – review and editing (equal). **Pauline Blaimont**: Data curation (equal); writing – review and editing (equal). **Pierre de Villemereuil**: Formal analysis (equal); validation (equal); writing – review and editing (equal). **Donald B Miles**: Conceptualization (equal); data curation (equal); validation (equal); writing – review and editing (equal). **Murielle Richard**: Conceptualization (equal); data curation (equal); project administration (equal); supervision (equal); validation (equal); writing – review and editing (equal). **Jean Clobert**: Conceptualization (equal); data curation (equal); project administration (equal); supervision (equal); validation (equal); writing – review and editing (equal).

## Supporting information

Appendix S1‐S5

Supplementary Material

## Data Availability

Data will be archived using a Mendeley deposit or as a Supporting Information file upon acceptance of the manuscript for publication.
