# Supplementary material for: Climate dependent heating efficiency in the common lizard
Source: Ecol Evol. 2020 Jul 16;10(15):8007–17. doi: 10.1002/ece3.6241 (PMC7417243; doi:10.1002/ece3.6241)
Supplement: Supplementary file 1 — Appendix S1‐S5 [file ECE3-10-8007-s001.docx]

**Appendix 1: Body Condition**

Body condition (BC) was defined as the residuals of a linear regression between body mass (response variable) and snout-ventral length (SVL; predictor variable). In many cases, body mass does not escalate linearly with body length, but rather follows a quadratic or cubic relationship. In our situation however, it appears that a linear relationship is enough to discriminate if a male common lizard is more or less corpulent than expected (See fig. S.1).


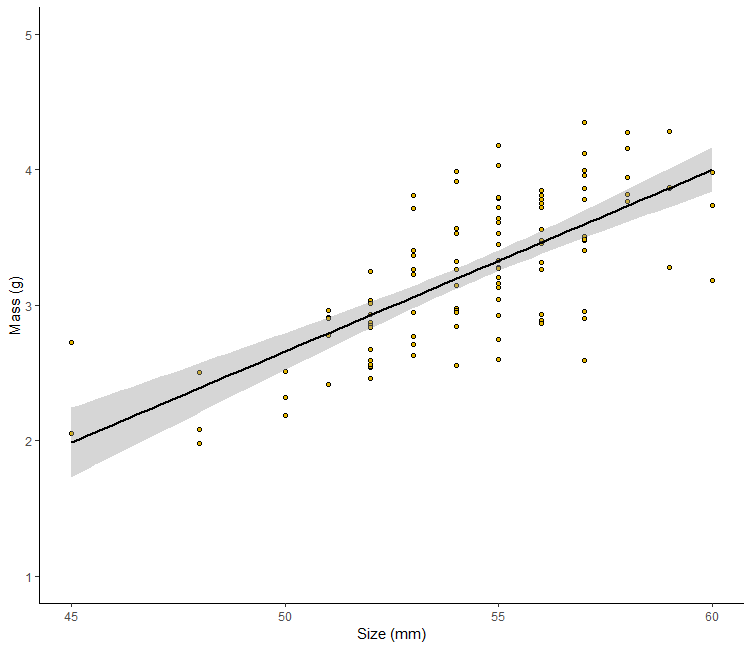


**Figure S.1.** Relationship between mass and SVL used here to characterize individuals body corpulence.

**Appendix 2 : Correlation between skin temperature and body temperature in the common lizard.**

Skin temperature recorded with an infrared thermometer is usually correlated with body temperature in small lizards, and thus considered as a good proxy of it. However, to confirm the relationship between dorsal skin temperature and body temperature, we measured the temperature of 20 lizards placed in different thermal conditions using two methods. First, as described in our experimental design, we measured the dorsal skin temperature with an infrared thermometer (model Fluke^®^568). We then compared this temperature with a cloacal temperature, measured immediately after the IR temperature, using a thermal probe. During the second measure, we minimize lizard manipulation not to influence the animal’s body temperature by heat transfer from experimenter’s hands. To cover a large range of body temperatures, we used different environment within the critical temperatures of the species [ ~10°C to 35°C]. Each lizard experienced two different environments and body temperatures were measured twice with each method in both environments. A total of 80 temperatures were recorded with each method.

We detected a correlation coefficient between IR temperatures and cloacal temperatures of 0.98. Nonetheless, it is interesting to note that for temperature under 30°C, dorsal skin temperatures seem lower than cloacal measurements in most of the case. On the contrary, above 30°C, IR temperatures seem to slightly overestimate cloacal temperatures. This effect however seems marginal and differences are generally low.

In conclusion, we think that infrared thermometer measured skin temperature is a good and valid proxy of intern body temperature for the common lizard.


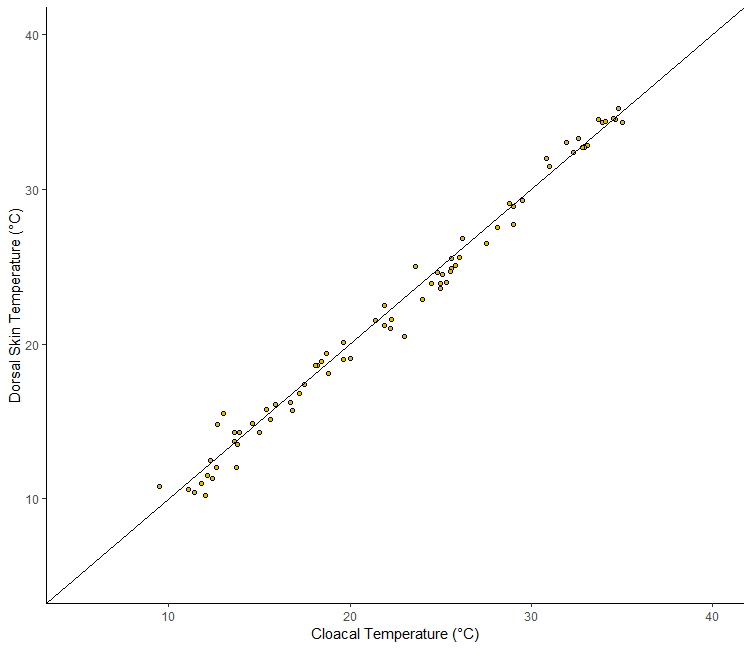


**Figure S.2.** Relationship between cloacal temperature and dorsal skin temperature for 80 common lzards placed in different thermal conditions.

**Appendix 3: Steady-state temperature**

Relation between the equilibrium temperature (*T_ss_*), predicted by the statistical model (see equation 1 in the main manuscript) and the body temperature recorded by each lizard at the end of the experiment. We found a correlation coefficient of 0.4, suggesting that higher *T_ss_* are found for individuals with warmer body temperatures at the end of the experiment.


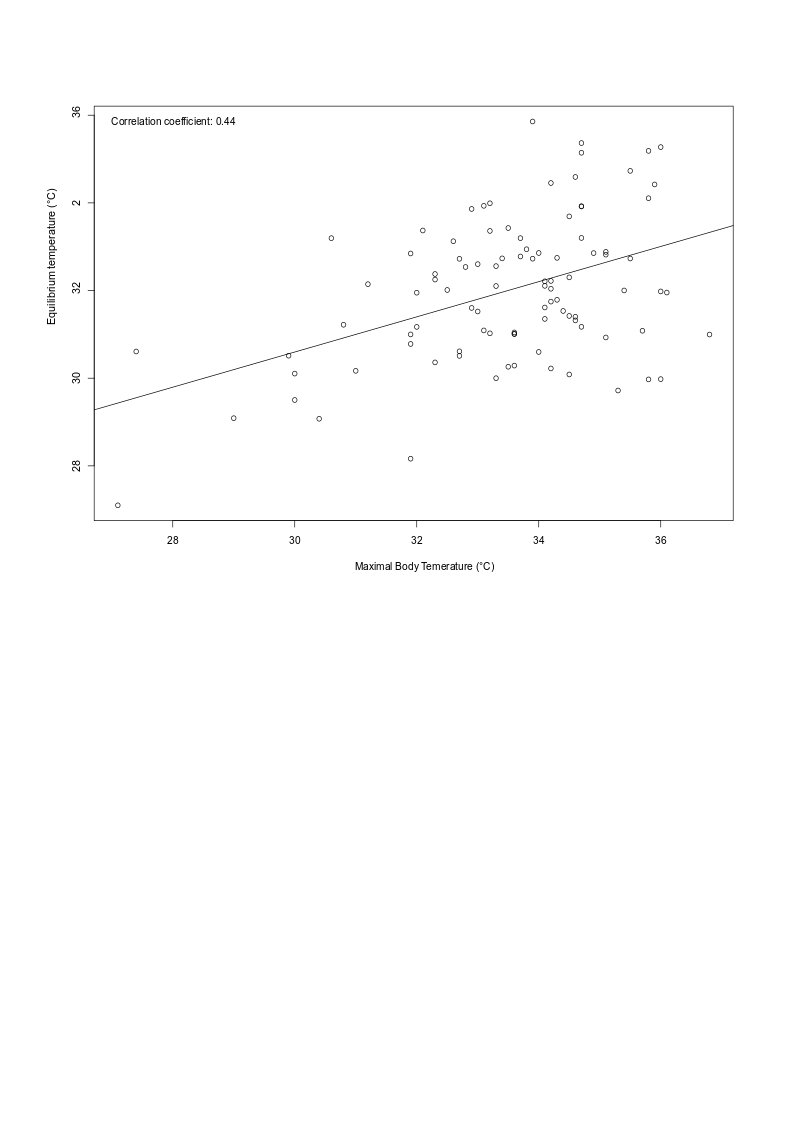


Steady-state temperature (°C)

Maximal body-Temperature (°C)

**Appendix 4: Patterns of precipitation**

Average daily precipitations we measured over 3 weeks have been compared to the rainfall conditions measured during the past 20 years by national weather forecast stations over the 20 years preceding our analyses (1995-2015). We detect a robust correlation between both set of values, suggesting that our sample of data represents well the weather conditions experienced by the studied populations.


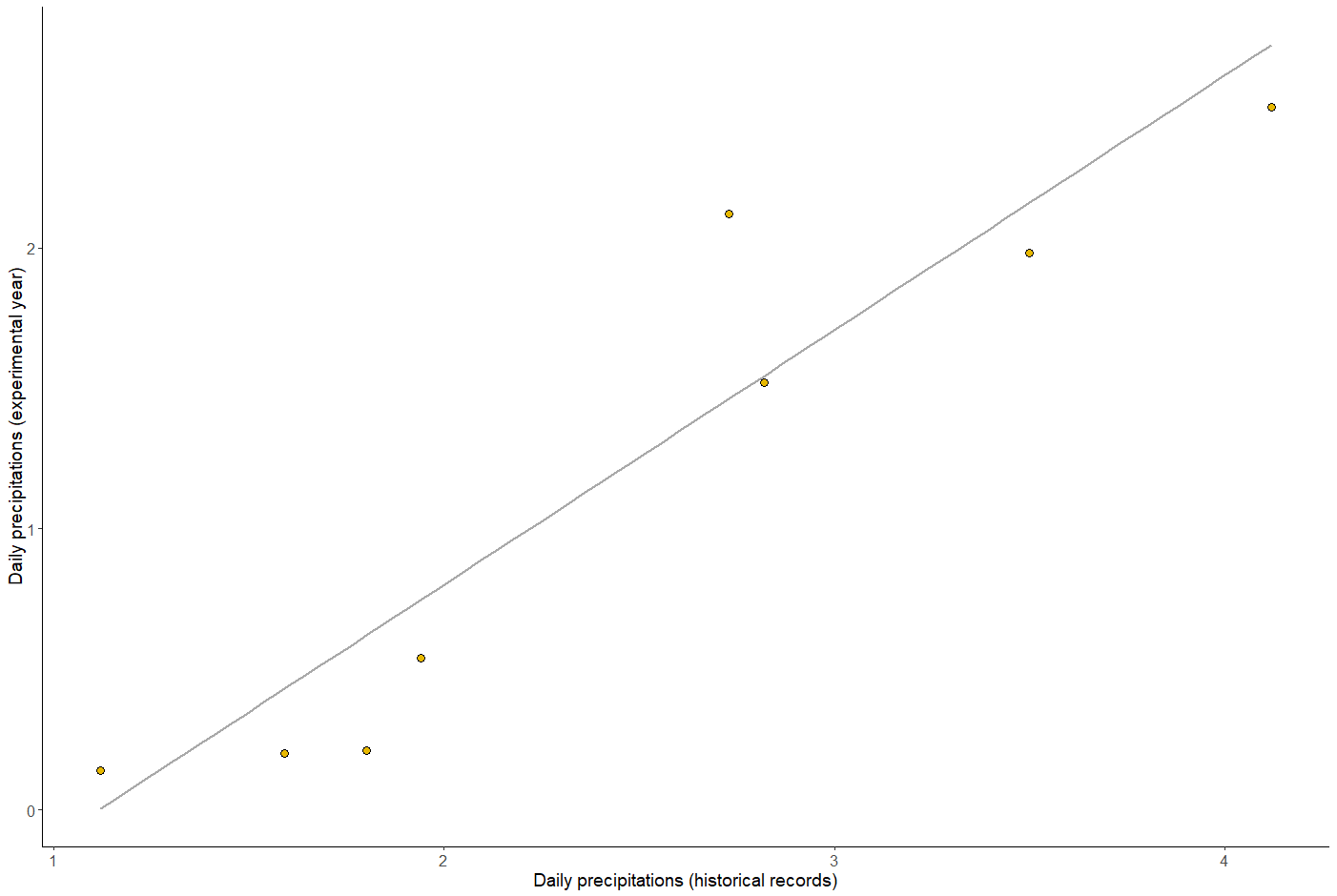


**Appendix 5: Cooling Rates**

Beside the heating experiment, we measured cooling rates. After heating, lizards were transferred to a holding plastic terrarium placed in a room at ambient laboratory temperature (T_lab_), recorded for use in statistical models. We estimated cooling rate by measuring dorsal temperature using the same infrared thermometer every five minutes for 20 minutes.

The statistical protocol used to analyze cooling rates is the same than describe for heating rate (see method section, in the main manuscript). In this case however, we mathematically approximated the cooling rates using a classical exponential decay curve where body temperature (*T_b_*) decreases according to time (*t*), initial temperature (*T_init2_*) and cooling rate (λ):

$T=T_{init2}e^{-\lambda t}$ (Eqn2, Fig. 2b)

Note that in statistical analyses, we also tested the influence of laboratory temperature (*T_lab_*) instead of tube temperature as lizards were not under the bulb anymore, and therefore subject to ambient temperatures. All continuous variables were mean-centered and scaled by the standard deviation.

|  |  |  | Predictor Variables | Estimate | SE | d.f. | t-value | *P*-value |
| --- | --- | --- | --- | --- | --- | --- | --- | --- |
|  |  |  |  |  |  |  |  |  |
|  | Cooling rate | λ | Intercept | -0.047 | 0.007 | 463 | -9.54 | <0.001***** |
|  |  |  | T_init_ | 0.002 | 0.0002 | 463 | 14.37 | <0.001***** |
|  |  |  | T_lab_ | -0.002 | 0.0004 | 463 | -7.95 | <0.001***** |
|  |  |  | Body Condition | -0.0003 | 0.0003 | 463 | -1.51 | 0.13 |
|  |  |  | Darkness | -0.0001 | 0.0004 | 463 | -0.72 | 0.46 |
|  |  |  | R_hab_ | 0.0002 | 0.0003 | 463 | 0.65 | 0.51 |
|  |  |  | T_Hmax_ | 0.0006 | 0.0002 | 463 | 2.98 | 0.03* |
|  |  |  | T_Hmin_ | 0.00004 | 0.0003 | 463 | 0.18 | 0.85 |
|  |  |  |  |  |  |  |  |  |
|  | Random effects (Id in Pop) | |  | SD | Residuals | |  |  |
|  |  |  | λ (intercept) | 6.88^e-08^ |  | 1.25 |  |  |

**Table S.4. Summary of non-linear mixed effect models** relating the thermal time constant for cooling rate (λ) vs. initial body temperate (T_init_), ambient lab temperature (T_lab_), body condition, dorsal darkness, habitat rainfall (R_hab_) and temperatures (T_Hmin,_ T_Hmax_ ). Note the smaller value of τ corresponds with a faster rate of heating. Individuals were nested within population as a random effect.


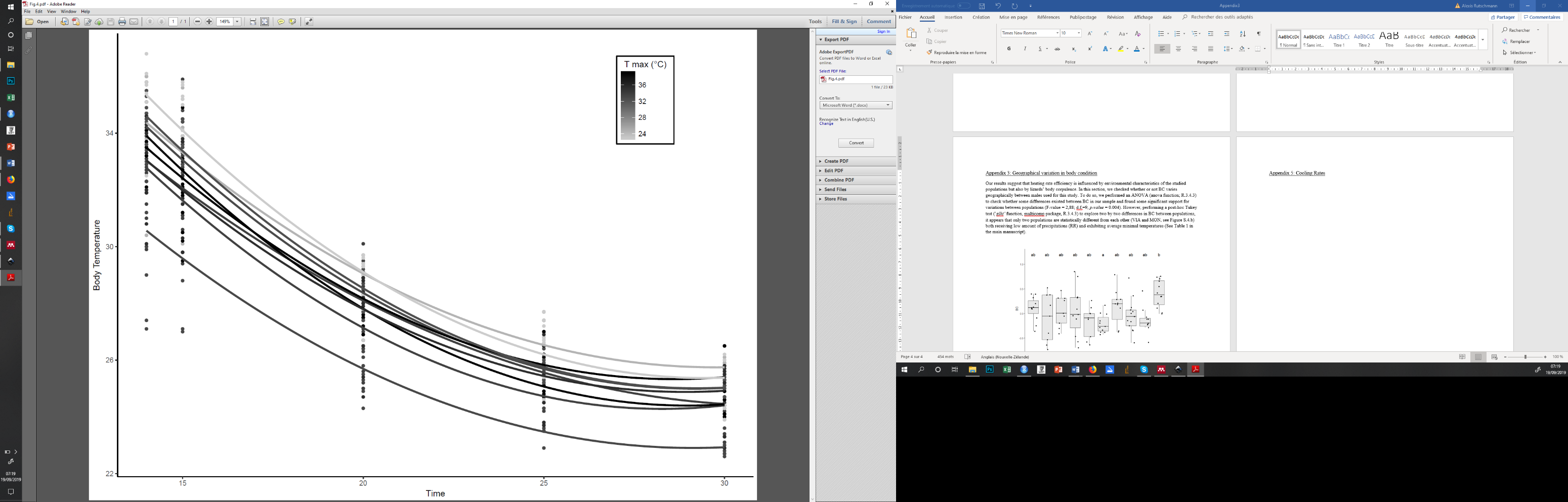


**Figure S.4. Heat loss versus environmental temperatures among populations.** Representation of skin surface temperature according to local maximal temperature (T_max_) in the studied populations. Dots represent individual measures. Solid lines represent an average heat curve for each population. A scale of the maximal temperature is provided in grey: the darker the coloration, the warmer the atmospheric temperature.

Results pointed out a positive effect of initial temperature *T_init_* on cooling rate *λ*; lizards with a high initial *T_b_* cooled down faster. However, higher ambient temperature (*T_lab_*) significantly buffered the rate of cool down as *T_lab_* negatively impacts *λ*. Neither dorsal darkness nor individual’s body condition significantly impacted λ. Finally, the observed rate of cooling appeared to positively dependent on the maximal temperatures recorded in the environment (T_Hmax_), with warmer temperatures inducing rapid cooling rates.
